# Lung Cancer Screening

**DOI:** 10.1155/DTE.2.i

**Published:** 1996

**Authors:** Yoshihiro Hayata

**Affiliations:** Tokyo Medical College Cancer Center Tokyo Japan


*Diagnostic and Therapeutic Endoscopy*, 1996, Vol. 2, p. i
Reprints available directly from the publisher
Photocopying permitted by license only

© 1996 Harwood Academic Publishers GmbH
Printed in Singapore

### Editorial: Lung Cancer Screening

YOSHIHIRO HAYATA

*Tokyo Medical College Cancer Center, Tokyo, Japan*

The review paper by Dr. Denis A. Cortese on "Lung Cancer Screening: Current Status in the United States" is an important description of the history and present situation of screening for lung cancer in the United States. Screening for lung cancer in the United States was halted after the three huge studies carried out in the 1970s by the Mayo Clinic, Memorial Sloan-Kettering Medical Center, and Johns Hopkins, because the results, although yielding a high detection rate of lung cancer, did not demonstrate a higher survival rate or cost-effective benefit.

Tokyo Medical College has been carrying out chest x-ray surveys for chest diseases on employees of the Tokyo Metropolitan Government since 1953, which makes it the longest continuously performed chest x-ray survey in the world. However, this is not a prospective randomized study, and the age of the oldest participants is limited by the retirement age of the metropolitan employees.

Looking at the cases of cancer detected in this group, 41.9% were stage I, 13.7% were stage II, and 44.4% were stages III and IV. The resectability rates were 18.3, 66.7, and 30.2%, respectively. A high-risk group was established approximately 20 years ago, consisting of those aged 45 or more and with a cigarette index (number of cigarettes per day times number of years smoking) of 400 or more, and these subjects were offered a semiannual survey. The rate of early-stage lung cancer detection in the semiannual group was 24.3%, compared with only 11.1% in the annual group and 10.3% in the group which had not received an examination in the previous year. These statistics show that, in the Japanese experience, screening certain high-risk groups is beneficial in terms of detection at an early stage, curative resection, prognosis, and also cost-beneficial treatment.

The Japanese experience does suggest that the studies described at the end of Dr. Cortese's paper will in fact show that chest x-rays are valuable in lung cancer screening and may even lead to attempts to use bronchoscopy as a screening method in certain very high-risk groups. In the case of gastric and esophageal cancer, the idea of endoscopic screening was initially rejected, but later studies showed that it is a valid approach, given an appropriate risk factor. It may well be that the studies described by Dr. Cortese will lead to an endoscopic approach to screen subjects at particularly high risk of developing lung cancer.

